# Re: Olivero A, et al. Adrenocortical carcinoma with venous tumor invasion: is there a role for mini-invasive surgery?

**DOI:** 10.1007/s00423-023-02827-2

**Published:** 2023-02-13

**Authors:** Marina M. Tabbara, Javier Gonzalez, Gaetano Ciancio

**Affiliations:** 1 Department of Surgery, University of Miami Miller School of Medicine, Miami, FL USA; 2 Miami Transplant Institute, University of Miami Miller School of Medicine, Jackson Memorial Hospital, 1801 NW 9th Ave, 7th Floor, Miami, FL 33136 USA; 3 Servicio de Urología, Unidad de Trasplante Renal, Hospital General Universitario Gregorio Marañón, Madrid, Spain; 4 Department of Urology, University of Miami Miller School of Medicine, Miami, FL USA

**Keywords:** Adrenal tumor, Tumor thrombus, Venous thrombus, Classification

## Abstract

**Purpose:**

The surgical treatment for adrenocortical carcinoma with venous tumor invasion remains a challenge for surgeons. A critical factor in determining the surgical approach is utilizing a classification system that accurately defines the tumor thrombus level.

**Methods:**

Olivero and colleagues report their experience regarding the feasibility of mini-invasive surgery for adrenocortical carcinoma with venous tumor invasion. They studied the outcome of 20 patients from 4 international referral center databases.

**Results:**

They describe a classification for adrenal tumor with tumor thrombus into four levels: (1) adrenal vein invasion; (2) renal vein invasion; (3) infra-hepatic inferior vena cava (IVC); and (4) retro-hepatic IVC.

**Conclusions:**

We congratulate the authors for their work and patient outcomes; however, in efforts to avoid confusion in the surgical community, we believe their classification system requires modification compared to our classification system developed in 2004.

Dear Prof. Dr. Markus W. Büchler,

Olivero and colleagues report their experience regarding the feasibility of mini-invasive surgery for adrenocortical carcinoma with venous tumor invasion [1]. They studied the outcome of 20 patients from 4 international referral center databases. Their conclusion was that mini-invasive approach for adrenal tumors with tumor thrombus is feasible in some patients. They also describe a classification for adrenal tumor with tumor thrombus into four levels: (1) adrenal vein invasion; (2) renal vein invasion; (3) infra-hepatic inferior vena cava (IVC); and (4) retro-hepatic IVC. We congratulate the authors for their work and patient outcomes; however, we believe they should report their classification as a modification of our previously reported classification [2] to avoid further confusion.

We first reported a classification system of adrenal tumor with tumor thrombus in 2004 [2]. We also recently reported the outcome of 10 patients with adrenal tumor with tumor thrombus, which is one of the largest series of adrenal tumor with tumor thrombus from a single institution and by a single surgeon [3]. Right adrenal tumor thrombus venous extension is different from left adrenal tumor with tumor thrombus venous extension. The right adrenal vein goes directly into the retro-hepatic inferior vena cava. According to our classification system, the right adrenal tumor **level I** tumor thrombus extends to the adrenal vein and/or infra-hepatic inferior vena cava (IVC); **level Ia** left adrenal tumor with tumor thrombus extending into the adrenal vein and/or renal vein; **level Ib** left adrenal tumor with tumor thrombus extending into the infra-hepatic IVC; **level II** right or left adrenal tumor with tumor thrombus in the hepatic portion of the IVC reaching to the level of the major hepatic veins and perhaps extending into them; **level IIIa** supra-hepatic and infra-diaphragmatic tumor thrombus extending into the retro-hepatic IVC above major hepatic veins but below the diaphragm; **level IIIb** supra-diaphragmatic and infra-atrial tumor thrombus extending into the supra-diaphragmatic intra-pericardial IVC, but not into the right atrium; and **level IV** the tumor thrombus extends into the right atrium. Level IIIa, level IIIb, and level IV can be associated with Budd-Chiari syndrome caused by the obstruction of the major hepatic veins by the tumor thrombus [2].

## References

[1] Olivero A, Liu K, Checchucci E, Liu L, Ma L, Wang G et al (2023) Adrenocortical carcinoma with venous tumor invasion; is there a role for mini-invasive surgery? Langenbecks Arch Surg 10:408(1):17. 10.1007/s00423-023-02765-z. 10.1007/s00423-023-02765-z 36625975

[2] Ekici S, Ciancio G (2004). Surgical management of large adrenal masses with or without thrombus extending into the inferior vena cava. J Urol.

[3] Ciancio G, Farag A, Gonzalez J, Gaynor JJ (2021). Adrenal tumors of different types with or without tumor thrombus invading the inferior vena cava: an evaluation of 33 cases. Surg Oncol..

